# Effect of plasma free fatty acid supply on the rate of ceramide synthesis in different muscle types in the rat

**DOI:** 10.1371/journal.pone.0187136

**Published:** 2017-11-02

**Authors:** Piotr Zabielski, Agnieszka Urszula Błachnio-Zabielska, Beata Wójcik, Adrian Chabowski, Jan Górski

**Affiliations:** 1 Department of Physiology, Medical University of Białystok, Białystok, Poland; 2 Department of Medical Biology, Medical University of Białystok, Białystok, Poland; 3 Department of Hygiene, Epidemiology and Metabolic Disorders, Medical University of Białystok, Białystok, Poland; 4 Medical Institute, Łomża State University of Applied Sciences, Łomża, Poland; Michigan State University, UNITED STATES

## Abstract

Ceramide is a key compound in sphingolipid metabolism. Dynamics of ceramide synthesis is important in the several biological processes, such as induction of apoptosis or insulin resistance. So far, its de novo synthesis rate was evaluated indirectly, based on the content of the compound, its intermediates and the activity of respective enzymes. The aim of the present study was to directly measure ceramide synthesis rate (FSR) in different muscle types under varied plasma FFA supply in rat with the use of [U-^13^C] palmitate tracer and LC/MS/MS. The experiments were carried out on male Wistar rats, divided into three groups: 1-control, 2-with elevated plasma free fatty acid (FFA) concentration by means of intralipid and heparin, 3-with reduced plasma FFA concentration by means of nicotinic acid. The stable plasma FFA concentration and plasma [U-^13^C] palmitate enrichment was maintained for two hours by simultaneous infusion of the tracer and the respective compounds. At the end of the experiment, samples of blood from the abdominal aorta, the heart, diaphragm, soleus and white section of the gastrocnemius were taken. Muscle sphinganine, sphingosine and ceramide content and enrichment and plasma palmitate enrichment was measured with the use of LC/MS/MS. Plasma FFA concentration and composition was measured by means of gas-liquid chromatography. Under basal conditions ceramide FSR in the heart and the diaphragm was higher than in the soleus and the white gastrocnemius. Elevation in the plasma FFA concentration increased the FSR and ceramide content in each muscle, which correlated with increased HOMA-IR. The highest FSR was noted in the heart. Reduction in the plasma FFA concentration decreased ceramide FSR in each muscle type, which was accompanied by marked reduction in HOMA-IR. It is concluded that ceramide FSR depends on both the muscle type and the plasma FFA supply and is correlated with whole body insulin sensitivity under varying plasma FFA supply.

## Introduction

**Fig 1:**
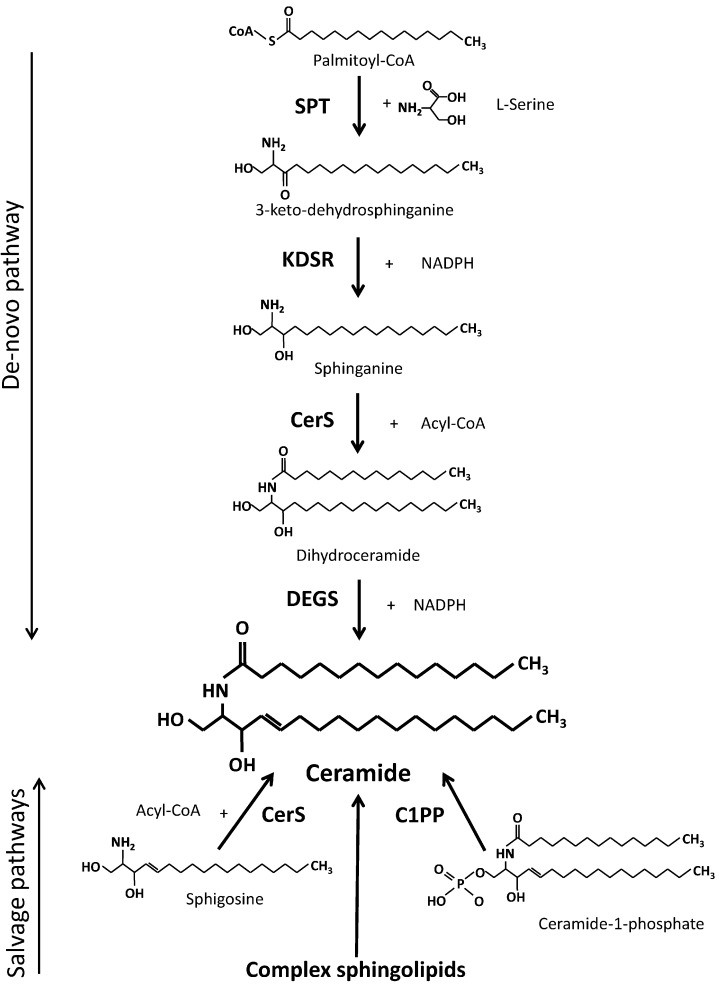
Ceramide synthesis pathways. SPT—serine palmitoyltransferase, KDSR—3-keto-dehydrosphinganine reductase, CerS—ceramide synthase (various isoforms), DEGS—dihydroceramide desaturase (various isoforms), C1PP—ceramide-1-phosphate phosphatase, Acyl-CoA—fatty acyl coenzyme A, NADPH—nicotinamide adenine dinucleotide phosphate, reduced.

Ceramide is the key compound in metabolism of sphingolipids. It consists the core of all complex sphingolipids and acts as a precursor of sphingosine and ceramide-1-phosphate [1, 2]. It should be also added that ceramide is very active biologically. Its major effects include pro-apoptotic, pro-inflamatory and anti-proliferative activities [3–5]. It also contributes to development of insulin resistance [6, 7]. There are four sources of ceramide (Fig 1): 1- de novo synthesis, 2- liberation from complex sphingolipids, 3- acylation of sphingosine and 4—dephosphorylation of ceramide-1-phosphate [1, 2]. *De novo* pathway of ceramide synthesis is the most important one, since it is also the source of all derivatives of ceramide including the complex sphingolipids. The 2–4 sources are secondary ones, since ceramide had to be first incorporated in these compounds. Those sources can be regarded as salvage pathways of ceramide generation, where ceramide or sphingosine backbone comes from complex sphingolipids. Detailed description of ceramide synthesis and salvage pathways are presented in the Fig 1.

Factors regulating metabolism of ceramide, including the activity of principal enzymes involved in the process were widely studied in different tissues. Long-chain fatty acids are needed in two steps of the pathway (Fig 1). Supply of long-chain fatty acids should be expected to play a key role in the determination of the rate of ceramide synthesis and quantity of ceramide generated by the de novo synthesis pathway. Regarding skeletal muscles, the data obtained in vitro in cultured C2C12 and L6 myocytes show that acute elevation in supply of fatty acids increases the content of ceramide [8–11]. In vivo, acute elevation in plasma FFA supply was found to increase the content of ceramide in the soleus [12] and the heart [13] of the rat and in human vastus lateralis muscle [14]. The latter observation was not confirmed in another study [15]. Interestingly, marked reduction in the plasma free fatty acid concentration did not affect the content of ceramide in human vastus lateralis [15]. Hu et al. [11] examined effect of excess of palmitate on ceramide metabolism in C2C12 myotubes using ^13^C-labeled palmitate. They found that the excess of palmitate increased the content of sphinganine and ceramide severalfold. This effect was blocked by inhibition of SPT thus indicating increased *de novo* synthesis of ceramide in the presence of the palmitate excess. So far, to our knowledge, the rate of ceramide *de novo* synthesis was only indirectly evaluated basing on activity of the enzymes involved in ceramide metabolism, the content of ceramide and intermediates of its metabolism. The aim of the present study was to examine ceramide fractional synthesis rate (FSR) in different rat muscles by the measurement of the incorporation rate of intravenously infused [U-^13^C]palmitate into muscular palmitoyl-ceramide. Moreover, we determined the effect of increased or reduced level of plasma free fatty acids on the synthesis rate and ceramide molecular species in heart, diaphragm, soleus and white section of the gastrocnemius. Our study for the first time describes the effect of varied fatty acids supply on both the ceramide synthesis rate and composition in rat muscles which differ by utilization of fatty acids.

## Materials and methods

### Animals

Experiments were carried out on 15 week old male Wistar rats (about 350g of body weight). Animals were acclimatized for one week prior to beginning of the experiment and housed on standard laboratory rat chow in controlled environment under ambient temperature and humidity in 12h light/dark cycle. One day before experiment animals were randomly assigned to 3 experimental groups (n = 10 each): control animals (C), treated with intalipid/heparin infusion (I+H), treated with nicotinic acid infusion (NA). Appropriate treatments for each of the experimental groups are given in “Infusion protocol” methods section. Experiments were approved by Institutional Animal Care and Use Committee of Medical University of Bialystok.

### Infusion protocol

**Fig 2:**
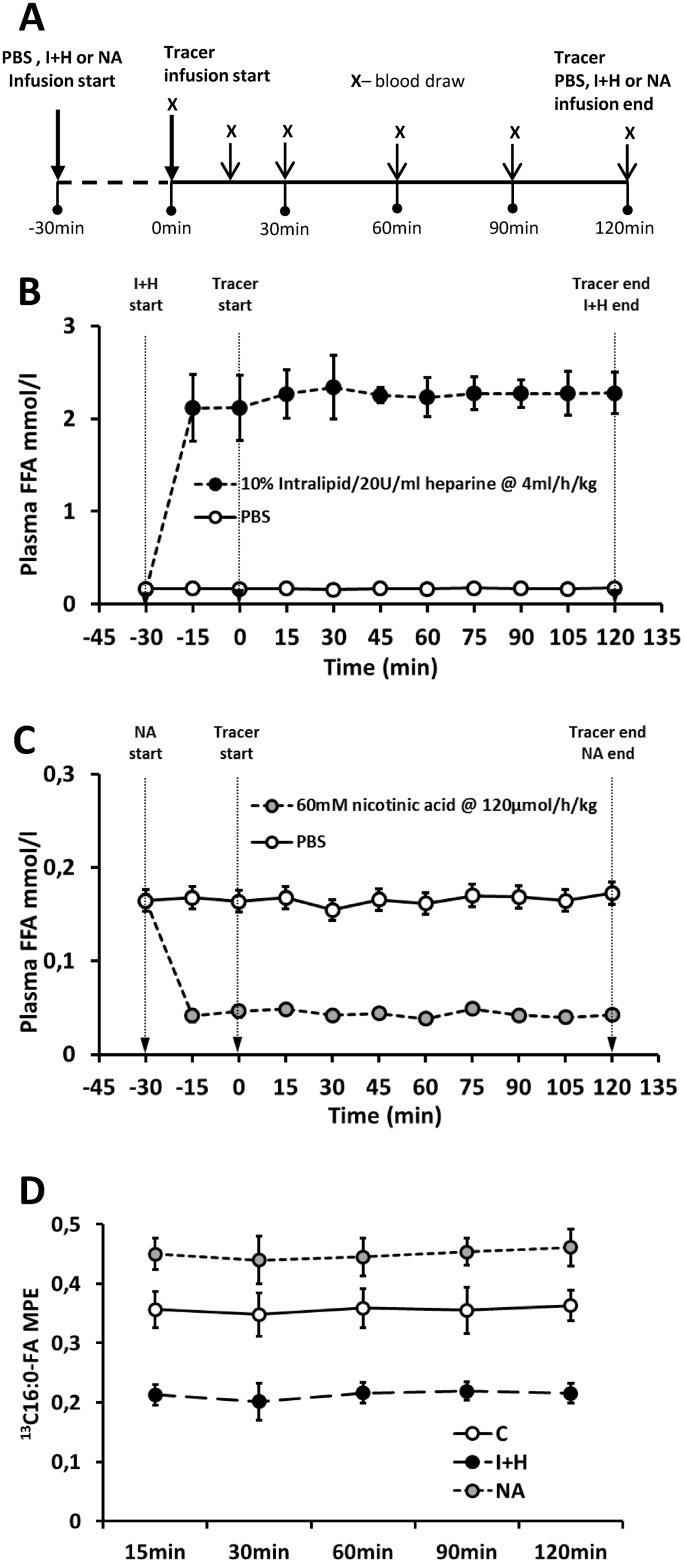
Infusion protocol and the effects of intralipid and heparin or nicotinic acid infusion on plasma free fatty acids concentration and palmitate enrichment in rats. (A)—outline of the infusion protocol; (B)–effect of intralipid and heparin infusion (n = 3 per group); (C)–effect of nicotinic acid infusion (n = 3 per group), (D)–plasma [U-^13^C]palmitate enrichment (n = 10 per group); C—control with phosphate buffered saline (PBS) infusion (grey circles); I+H—intralipid and heparin infusion (closed circles); NA—nicotinic acid infusion (grey circles). Values represent mean +/- SD.

Infusion of intralipid, nicotinic acid and labeled palmitate was based on the methods by Hagman et al [16], Oh et al. [17] and Blachnio-Zabielska et al. [18], respectively. Uniformly labeled [U-^13^C]-palmitate (Sigma-Aldrich, St. Louis, MO, USA) was prepared in fatty acids free albumin (Sigma-Aldrich) as albumin-bound potassium salt according to Guo et al.[19]. Infusate was adjusted to a final concentration of 1mM, filter-sterilized with 0.2μm syringe filter and stored in -20°C until use. The outline of infusion experiment is presented in Fig 2A. The food was withdrawn 3 hours prior to infusion. Animals received pentobarbital anesthesia intraperitoneally at a dose of 50mg/kg and were placed on a heating blanket. Tail was shaved off and tail blood circulation was stimulated by a heating lamp. Elastic infusion catheter (MTV 1, Braintree Scientific, Braintree, MA) was inserted through integrated 23Ga needle into lateral tail vein and the catheter was secured with small drop of cyanoacrylic adhesive and piece of fabric tape. Infusion line was connected to two syringe pumps (NE-1000 model, New Era Pump Systems, Farmingdale, NY) through Y connector. To obtain stable level of fatty acids in plasma for calculation of steady-state FFA kinetics, 30 minutes prior to tracer infusion H+I animals received 0.7ml/kg bolus of 10% Intralipid (Fresenius Kabi AB, Bad Homburg, DE) with 20U/ml of heparin (Merck, Darmstadt, DE), followed by continuous infusion at a rate of 4ml/kg/h. Similarly, NA treated animals received primed, continuous infusion of 30mM nicotinic acid (Calbiochem, San Diego, CA) in sterile phosphate buffered saline (20μmol/kg bolus, 120μmol/kg/h continuous) 30 minutes prior to tracer infusion. Control animals received infusion of phosphate buffered saline. Representative plasma free fatty acids level during phosphate buffered saline (control), intralipid+heparin or nicotinic acid infusion is presented in the Fig 2A and 2B (as measured by Free Fatty Acid Quantification Fluorometric Kit, BioVision, Milpitas, CA). After initial 30 minutes, animals received bolus of [U-^13^C]palmitate tracer at 0.5 μmol/kg to prime palmitate pool and accelerate isotopic equilibration, followed by continuous infusion at 3μmol/kg/h for the next 2 hours. To prevent increase in total body fluids, the infused volume did not exceeded 17% of total plasma volume (calculated according to Lee et al. [20] and was lower than 2-hour urine output, according to the values for Wistar rats from Rat Phenome Database [21]. At 0, 15, 30, 60 and 90 minutes of tracer infusion 50μl blood sample was taken from saphenous vein into heparinized Microvette capillary tube (Stardstedt, Numbrecht, Germany) to estimate both the concentration and enrichment of the palmitate in plasma by LC/MS. At the end of 2 hour infusion, the final blood sample was collected from inferior vena cava and rats were sacrificed by heart incision. The Fig 2C presents enrichment of plasma palmitate during respective infusion protocols (as estimated with liquid chromatography, tandem mass spectrometry). The samples of ventral diaphragm, left ventricle of the heart, soleus and white gastrocnemius muscle were collected and immediately frozen in liquid nitrogen for further analysis.

### Plasma free fatty acids

Plasma free fatty acids content and composition in the final blood sample were measured by gas-liquid chromatography, according to Nawrocki et al. [22]. Briefly, lipids were extracted from 100μl of plasma with chloroform/methanol solution (2:1, v/v) containing 0.05% of butylated hydroxytoluene (BHT, antioxidant, Sigma-Aldrich) and heptadecanoic acid (C17:0 FA, Sigma-Aldrich) as internal standard. Organic phase was washed with H_2_O, separated by centrifugation and resolved by thin-layer chromatography (TLC) with the use of heptane/isoprophyl ether/acetic acid (60:40:3; v/v/v) solvent mixture. Fractions corresponding to free fatty acids were scraped off and methylated with 14% boron trifluoride in methanol (Sigma-Aldrich) for 2 minutes at 100°C. Individual fatty acid methyl esters were separated on a CP-Sil 88 column (50 m, 0.25 mm ID, 0.20 μm film) with the use of Agilent 5890 Series II chromatograph. Individual FA concentration was measured against standard curves prepared on commercially available fatty acids standards (Larodan Fine Chemicals, Malmo, SE).

### Plasma insulin and glucose concentration and HOMA-IR calculation

Plasma insulin and glucose concentration was measured in the blood sample collected at the end of the infusion protocol with Mouse Insulin ELISA Kit (Mercodia AB) and Glucose Fluorometric Assay Kit (Sigma-Aldrich), respectively. Estimation of homeostasis model assessment of insulin resistance (HOMA-IR) was calculated according to Cacho et al [23] with the use of the following equation:

$$HOMA-IR\;=\;\frac{\left(Plasma\;glucose\;(mg\times {dl}^{-1})\times Plasma\;insulin\;(\mu U\times {ml}^{-1})\right)}{2430}$$

### Plasma palmitate concentration and enrichment

Plasma palmitate enrichment was measured according to Persson et al. [24], with the use of Agilent 1290 UHPLC and Agilent 6460 triple quadrupole mass spectrometer. Briefly, 250μl of plasma was spiked with internal standard (50μl of heptadecanoate, 60μg/ml in 2% albumin in PBS). Fatty acids were extracted with acidic Dole solution (isopropyl alcohol:heptane:1N H2SO4; 40:10:1; v/v/v), evaporated to dryness under nitrogen and re-suspended in 80% acetonitrile/0.5 mM ammonium acetate. Palmitate and heptadecanoate were separated on Zorbax C18, 1.8 μm, 2.1 ×150 mm column (Agilent) with appropriate buffer system (Buffer A: 80% acetonitrile/0.5 mM ammonium acetate; Buffer B: 99% acetonitrile, 1% 0.5 mM ammonium acetate). Negative ESI ionization and SIM mode was used to monitor ions corresponding to palmitate [M+2-H], [U-^13^C]palmitate [M+16-H] and heptadecanoate [M+2-H]. Plasma palmitate concentration and isotopic enrichment was calculated with the use of appropriate concentration and enrichment curves prepared on commercially available standards (Sigma-Aldrich).

### Tissue sphingolipids concentration and ceramide enrichment

Muscle sphingosine, sphinganine and ceramide content and isotopic enrichment of palmitoyl-ceramide was measured according to Blachnio-Zabielska et al. [24] with minor modifications. Briefly, tissue samples were pulverized in LN_2_, spiked with internal standards (d17:1-Sph, d17:1-Spa, d17:1-S1P, C17:0-Cer and C25:0-Cer, Avanti Polar Lipids Alabaster, AL) and homogenized on ice in cold homogenization buffer (50mM Tris/HCl, pH 7.4, 0.25M sucrose, 25mM KCl and 0.5mM EDTA). After sonication for 30s on ice, the homogenate was extracted twice with 2-propanol/water/ethyl acetate (30/10/60; v:v:v). Organic phase was evaporated under nitrogen and re-suspended in Buffer A and sphingolipids were resolved on Zorbax C8, 1.8μm, 2.1 ×150mm column with the use of Agilent 1290 UHPLC and two buffer system (Buffer A: 2mM ammonium formate, 0.1% formic acid in LC/MS grade methanol, Buffer B: 1mM ammonium formate, 0.1% formic acid in water). Each sample was analyzed in two runs: first for concentration measurement and the second one for enrichment measurement. Eluting sphingolipids were monitored on Agilent 6460 MS in +ESI mode as [M+H]+. In second run C16:0-Cer and [^13^C_16_]16:0-Cer were monitored as [M+2+H]+ and [M+16+H]+ ions respectively. The analyzes were performed in MRM mode. Concentration of sphingosine, sphinganine and individual ceramides and isotopic enrichment of palmitoyl-ceramide was calculated with the use of calibration curves prepared on commercially available standards (Avanti Polar Lipids Alabaster, AL) and [^13^C_16_]16:0-Cer synthesized by Lipidomics Core, MUSC (Medical University of South Carolina).

### Calculation of plasma fatty acids turnover rate and ceramide fractional synthesis rate

Each of the protocols (C, I+H, NA) resulted in stable plasma fatty acid concentration during 2 hours of the infusion (Fig 2B and 2C). Primed continuous infusion of labeled palmitate produced quick isotopic equilibration in each of the experimental groups (Fig 2D), thus the rate of appearance of palmitate (palmitate Ra) was calculated using the steady-state equations:

PalmitateRa=[U-C13]palmitateinfusionrate(nmolmin/kg)Meanplasma[U-C13]palmitateenrichment(MPE)

Total fatty acid Ra (FFA Ra) was calculated by dividing the palmitate Ra by the proportional contribution of palmitate the total concentration fatty acids as measured by gas-liquid chromatography (GC). After reaching steady-state plasma fatty acids rate of disappearance (FFA Rd) balanced plasma fatty acids rate of appearance (FFA Ra), thus FFA Ra was equal to FFA turnover rate.

Ceramide fractional synthesis rate was calculated by the tracer incorporation method according to Zhang et al. [25], which is based on the precursor-product principle:

$$Cer\;FSR\;(\%/h)\;=\;\frac{\left(CerE_{t2h}^{\;}-CerE_{t0}^{\;}\right)}{\int_{t0}^{t2h}EA\Delta t}$$

Where (*CerE*_*t*2_ − *CerE*_*t*1_) is the enrichment increment of ceramide-bound palmitate from the beginning (t0) to the end (t2h) of tracer infusion and $\int_{t0}^{t2h}EA\Delta t$ is the integral of enrichment versus time function (the area under plasma precursor enrichment curve) approximated using trapezoidal rule. Values *CerE*_*t*2h_, *CerE*_*t*0_ and $\int_{t0}^{t2h}EA\Delta t$ were calculated individually for each animal.

### Statistical analysis

Statistical significance was estimated using ANOVA with the Tukey honest significant difference post hoc test. Significance level was set to P < 0.05. Statistical significance was calculated between different infusion protocols within each of the muscle types and between the muscles within each of the infusion protocols. Correlation analysis was performed using Pearson’s r approach. To correct p-value for multiple comparisons we lowered initial p-value of Pearson’s r according to Bonferroni correction. For Cer FSR vs. individual ceramides correlation initial p-value of 0.05 was lowered to 0.0042. For plasma fatty acids vs muscle ceramides correlation, p-value was lowered to 0.00025. The results of correlation analysis for individual muscles are listed in supplement tables.

## Results

### Plasma free fatty acids concentration, turnover rate and insulin sensitivity parameters

**Fig 3:**
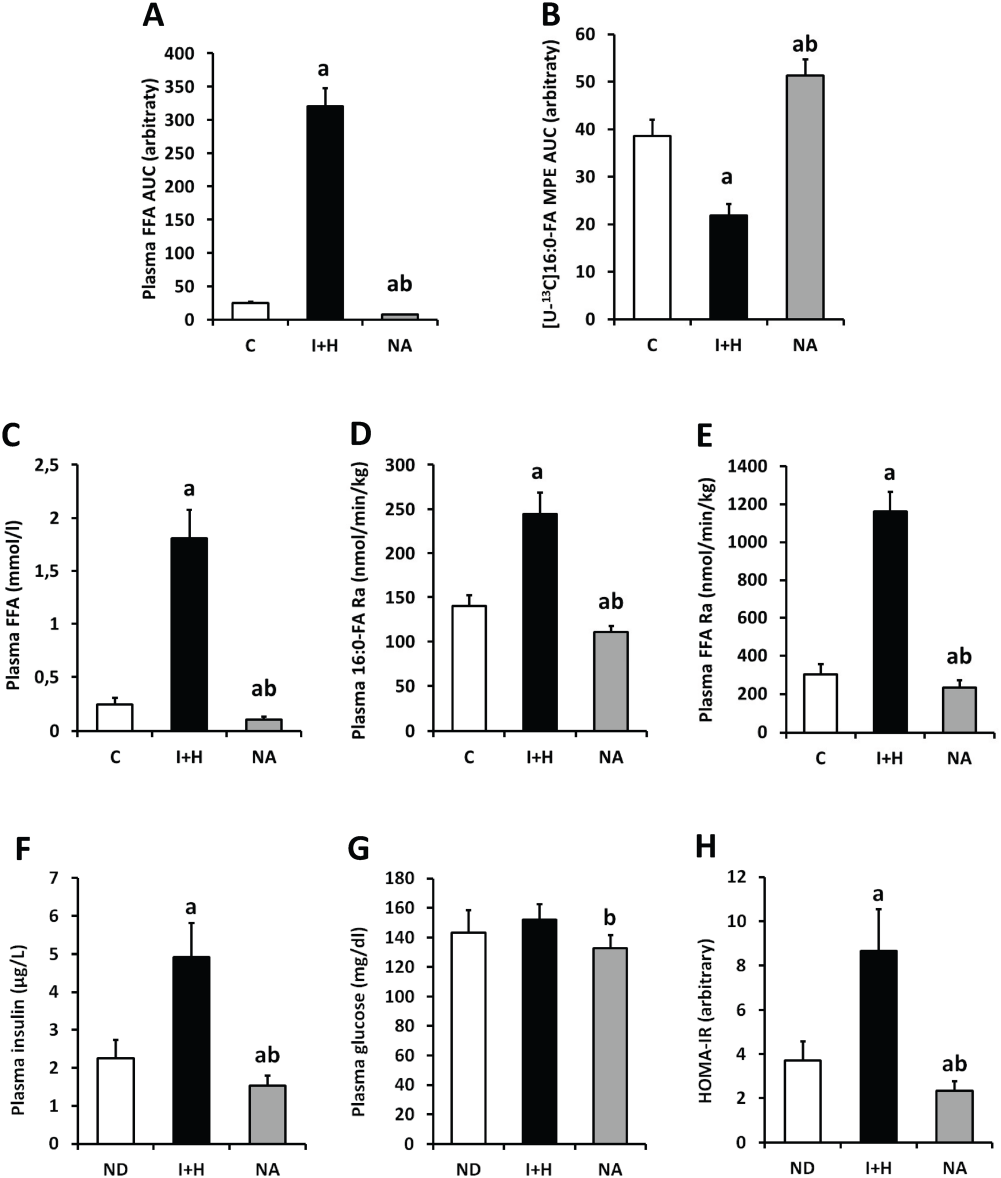
The impact of intralipid and heparin infusion or nicotinic acid infusion on plasma fatty acids, palmitate enrichment and insulin sensitivity parameters in rats. (A)–total area under total plasma fatty acids concentration from Fig 2B and 2C (FFA AUC, n = 3 per group); (B)–total area under plasma palmitate enrichment from Fig 2D (MPE AUC, n = 10 per group); (C)–total plasma FFA concentration (n = 10); (D)–plasma palmitate turnover rate (n = 10); (E)–total plasma FFA turnover rate (n = 10); (F)–Plasma insulin (n = 10); (G)–Plasma glucose (n = 10); (H)–Estimation of homeostasis model assessment of insulin resistance (HOMA-IR; n = 10). C—control group; I+H—intralipid + heparin group; NA—nicotinic acid group. Values represent mean +/- SD; a—p<0.05 vs C, b—p<0.05 vs I+H.

Plasma fatty acids supply increased several fold in intralipid+heparin group (I+H, Fig 3A and 3C), which significantly lowered plasma precursor enrichment pool (Fig 3B). Increase in plasma FFA was accompanied by significant elevation in both the free palmitate and total plasma fatty acids turnover rate (Fig 3D and 3E, respectively). Intralipid and heparin infusion increased both the plasma insulin level and HOMA-IR value, yet had no significant effect on plasma glucose concentration (Fig 3F, 3H and 3G, respectively). Decrease in the content of plasma fatty acids in nicotinic-acid treated animals was accompanied by moderate yet significant decline in both the palmitate and total plasma FFA turnover rate as compared to control. Interestingly, those parameters were accompanied by significant decrease in both insulin and glucose plasma concentration and strong decline in HOMA-IR value as compared to both the C and I+H groups.

### Plasma individual fatty acid concentration

The concentration of each fatty acid in I+H group increased as compared to the respective control value with the exception of C24:1 acid (Table 1). In NA group, the level of 14:0, 16:0, 16:1, 18:0, 18:2, 18:3, 20:5, 22:6, 24:1 fatty acids significantly decreased vs. the respective level in the control group.

**Table 1:** The effect of intralipid and heparin or nicotinic acid infusion on rat individual plasma fatty acids concentration.

FFA (nmol/l)	C		I+H		NA	
	Mean	SD	Mean	SD	Mean	SD
**14:0-FA**	8.30±1.13		12.65±2.34^a^		3.88±1.88^a^^b^	
**16:0-FA**	116.62±46.24		379.06±60.92^a^		50.49±14.63^a^^b^	
**16:1-FA**	18.95±6.91		36.97±9.64^a^		4.83±2.80^a^^b^	
**18:0-FA**	33.25±9.08		100.29±8.67^a^		18.05±3.70^a^^b^	
**18:1-FA**	33.53±9.08		338.37±54.09^a^		14.53±7.62^b^	
**18:2-FA**	17.02±5.01		784.01±114.66^a^		6.41±1.63^a^^b^	
**18:3-FA**	3.62±1.31		104.0±616.72^a^		0.48±0.16^a^^b^	
**20:0-FA**	0.38±0.08		3.37±0.72^a^		0.25±0.04^b^	
**20:4-FA**	3.26±1.11		13.79±3.42^a^		2.18±0.37^b^	
**20:5-FA**	1.32±0.53		8.64±4.91^a^		0.36±0.09^a^^b^	
**22:0-FA**	0.18±0.05		3.84±0.89^a^		0.14±0.05^b^	
**22:6-FA**	4.83±1.66		21.92±10.32^a^		1.32±0.31^a^^b^	
**24:0-FA**	0.76±0.21		2.35±0.26^a^		0.76±0.14^b^	
**24:1-FA**	0.20±0.02		0.17±0.03		0.14±0.03^a^^b^	
**Total FA**	**242.24**±63.15		**1809.48**±268.60^a^		**103.83**±27.25^a^^b^	

C- control; I+H—intralipid and heparin infusion; NA—nicotinic acid infusion

Values are mean +/- SD; n = 10 per group;

^a^- p<0.05vs. control;

^b^- p<0.05 vs I+H group

### The ceramide fractional synthesis rate and the total level of ceramide in different muscles

**Fig 4:**
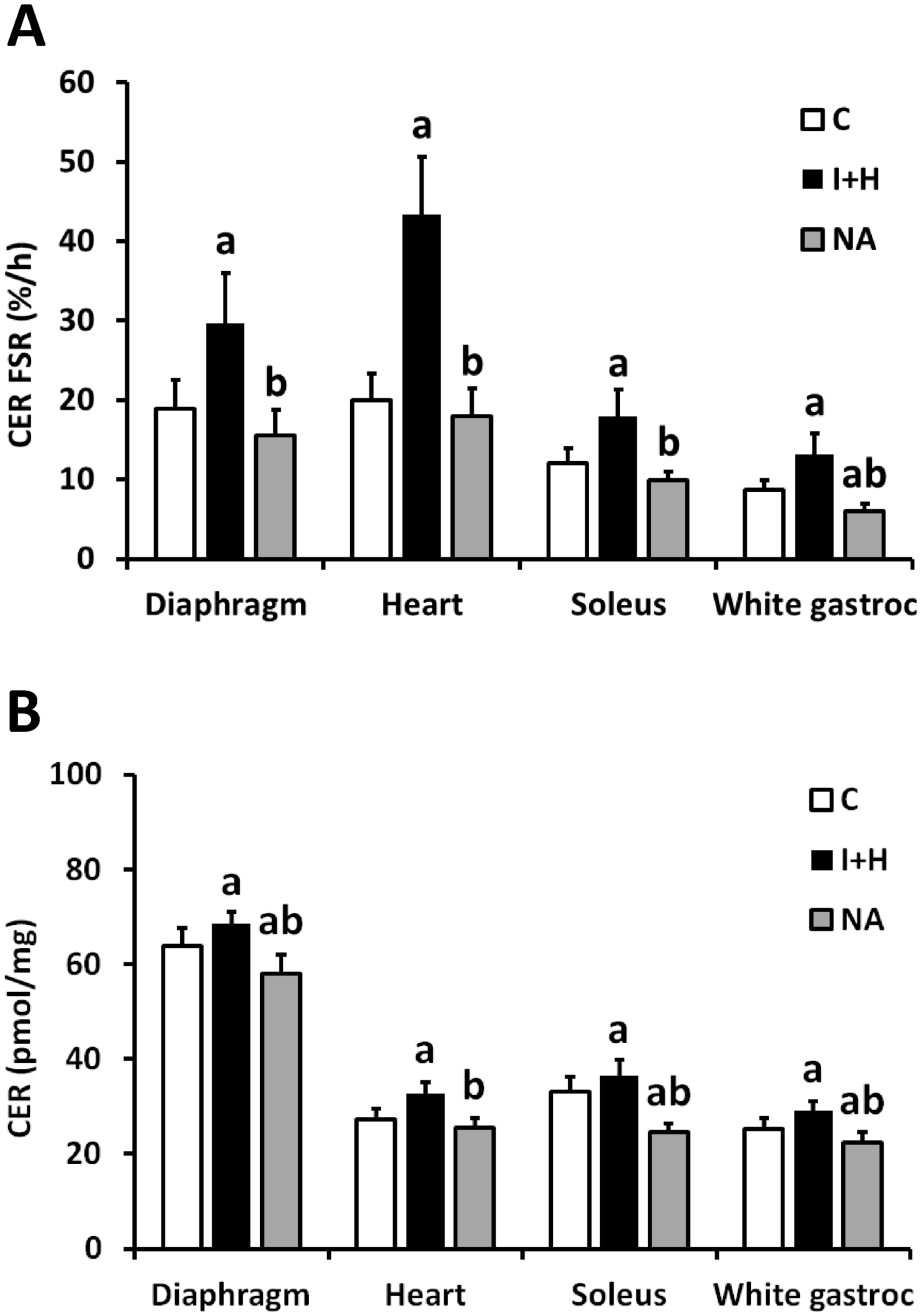
The impact of intralipid and heparin infusion or nicotinic acid infusion on ceramide fractional synthesis rate and ceramide content in different rat muscles. Panel A—ceramide fractional synthesis rate (CER FSR, n = 10); Panel B—total content of ceramide (CER, n = 10); C—control group; I+H—intralipid + heparin group; NA—nicotinic acid group. Values represent mean +/- SD; a—p<0.05 vs C, b—p<0.05 vs I+H.

In the control group, the ceramide fractional synthesis rate (Cer FSR) in the heart was almost equal to Cer FSR in the diaphragm (Fig 4A). Cer FSR in the white gastrocnemius was significantly lower than in the soleus. Cer FSR in either leg muscle was significantly lower than in the heart and in the diaphragm. In I+H group, Cer FSR in each muscle was significantly higher as compared the respective control value (Fig 4A). Highest Cer FSR value was noted in the heart, then diaphragm, then in soleus, whereas lowest Cer FSR was noted in white gastrocnemius. Increase in the content of plasma fatty acids differently affected ceramide FSR in each of the studied muscle. The highest increase (more than two fold) was observed in the heart. In other muscle types the ceramide FSR increased by 50% over the control value. Under nicotinic acid treatment, Cer FSR did not differ from the control value in diaphragm, heart and soleus muscle and was significantly lower than control value in the white gastrocnemius.

Elevation of plasma FFA in I+H groups led to modest, yet significant increase in ceramide content in all of the studied muscles (Fig 4B). The highest, 20% increase was noted in the heart, whereas lowest was noted for diaphragm (7% increase). Treatment with nicotinic acid significantly decreased the content of muscular Cer below the respective control value in diaphragm, soleus and white gastrocnemius. In the control group the total level of ceramide in the diaphragm was around two times higher than in the other muscles studied (p<0.05, Fig 4B). The level in the soleus was higher by 22% than in the heart (p<0.05) and by 33% than in the white gastrocnemius (p<0.05). Those reciprocal ratios were similar under I+H treatment, with soleus ceramide content still significantly higher than in the heart (by 11%, p<0.05) and white gastrocnemius (by 22%, p<0.05). Under nicotinic acid treatment there were no significant differences in ceramide content between heart, soleus and white gastrocnemius.

### The level of individual ceramide species in the muscles

In the heart of the I+H group the level of C22:0, C24:0 and C:24:1 ceramide was higher than the respective control level (Table 2). In the NA group the levels of individual ceramides did not differ from the control levels in the muscle.

**Table 2:** The effect of intralipid and heparin or nicotinic acid infusion on ceramide molecular species in rat diaphragm, heart, soleus and white gastrocnemius muscle.

Ceramide (pmol/mg)	Diaphragm						Heart						Soleus						White Gastrocnemius					
	C		I+H		NA		C		I+H		NA		C		I+H		NA		C		I+H		NA	
	Mean	SD	Mean	SD	Mean	SD	Mean	SD	Mean	SD	Mean	SD	Mean	SD	Mean	SD	Mean	SD	Mean	SD	Mean	SD	Mean	SD
**C14:0-Cer**	0.11±0.01		0.10±0.01		0.08±0.01^a^^b^		0.06±0.01		0.06±0.02		0.06±0.01		0.07±0.02		0.06±0.01		0.04±0.01^a^^b^		0.05±0.01		0.04±0.01		0.04±0.01	
**C16:0-Cer**	10.7±1.72		10.13±1.08		7.16±1.14^a^^b^		8.25±1.01		8.89±0.91		7.83±0.77		5.81±0.98		5.48±0.51		3.71±0.60^a^^b^		4.42±0.69		5.54±0.81^a^		4.02±0.55^b^	
**C18:0-Cer**	15.90±2.16		17.70±0.91		15.29±1.86^b^		3.11±0.41		3.41±0.49		3.10±0.28		12.89±1.67		16.02±2.83^a^		11.13±1.15^b^		10.72±1.31		12.76±1.60^a^		9.23±1.41^b^	
**C18:1-Cer**	0.29±0.03		0.31±0.04		0.29±0.04		0.03±0.01		0.04±0.00		0.04±0.01		0.16±0.02		0.18±0.03		0.16±0.02		0.25±0.05		0.27±0.06^a^		0.22±0.04^b^	
**C20:0-Cer**	5.45±0.67		5.45±0.54		4.94±1.07		5.86±0.80		6.89±1.80		5.70±0.84		1.92±0.28		1.80±0.25		1.09±0.20^a^^b^		0.73±0.10		0.86±0.13		0.68±0.10^b^	
**C22:0-Cer**	5.13±0.53		6.01±0.71^a^		4.85±0.72^b^		2.89±0.38		4.65±0.66^a^		2.60±0.29^b^		1.55±0.20		1.52±0.32		1.00±0.16^a^^b^		0.92±0.09		1.07±0.13		0.88±0.16^a^^b^	
**C24:0-Cer**	15.73±1.62		17.43±1.24^a^		14.19±1.79^b^		4.33±0.49		5.46±0.68^a^		3.83±0.57^b^		6.10±0.89		6.28±0.69		4.00±0.39^a^^b^		4.18±0.50		4.27±0.38		3.61±0.45^a^^b^	
**C24:1-Cer**	11.16±1.41		11.48±0.64		11.15±1.56		2.66±0.32		3.24±0.54^a^		2.33±0.26^b^		4.69±0.58		5.18±0.43		3.53±0.56^a^^b^		4.04±0.60		4.22±0.49		3.69±0.33	
**Total**	**63.10**±3.70		**68.61**±2.37^a^		**57.96**±4.13^a^^b^		**27.20**±2.30		**32.65**±2.38^a^		**25.48**±1.93^b^		**33.19**±3.08		**36.52**±3.41^a^		**24.67**±1.76^a^^b^		**25.32**±2.27		**29.04**±2.03^a^		**22.37**±2.21^a^^b^	

C- control; I+H—intralipid and heparin infusion; NA—nicotinic acid infusion

Values are mean +/- SD; n = 10 per group;

^a^- p<0.05vs. control;

^b^- p<0.05 vs I+H group;

In the diaphragm of the I+H group the level of C22:0 and C24:0 ceramide was higher whereas in the NA group the level of C14:0 and C16:0 ceramide was lower from the appropriate control level.

In the soleus, in I+H group the level of C18:0 ceramide was higher than the control level. In NA group, the level of each ceramide with the exception of C18:0 andC18:1 ceramide was lower from the control value.

In the white gastrocnemius of the I+H group the level of C16:0 and C18:0 ceramide increased and in NA group the level of C22:0 and C24:0 ceramide decreased vs. their own controls.

### The level of sphinganine and sphingosine in the muscles

**Fig 5:**
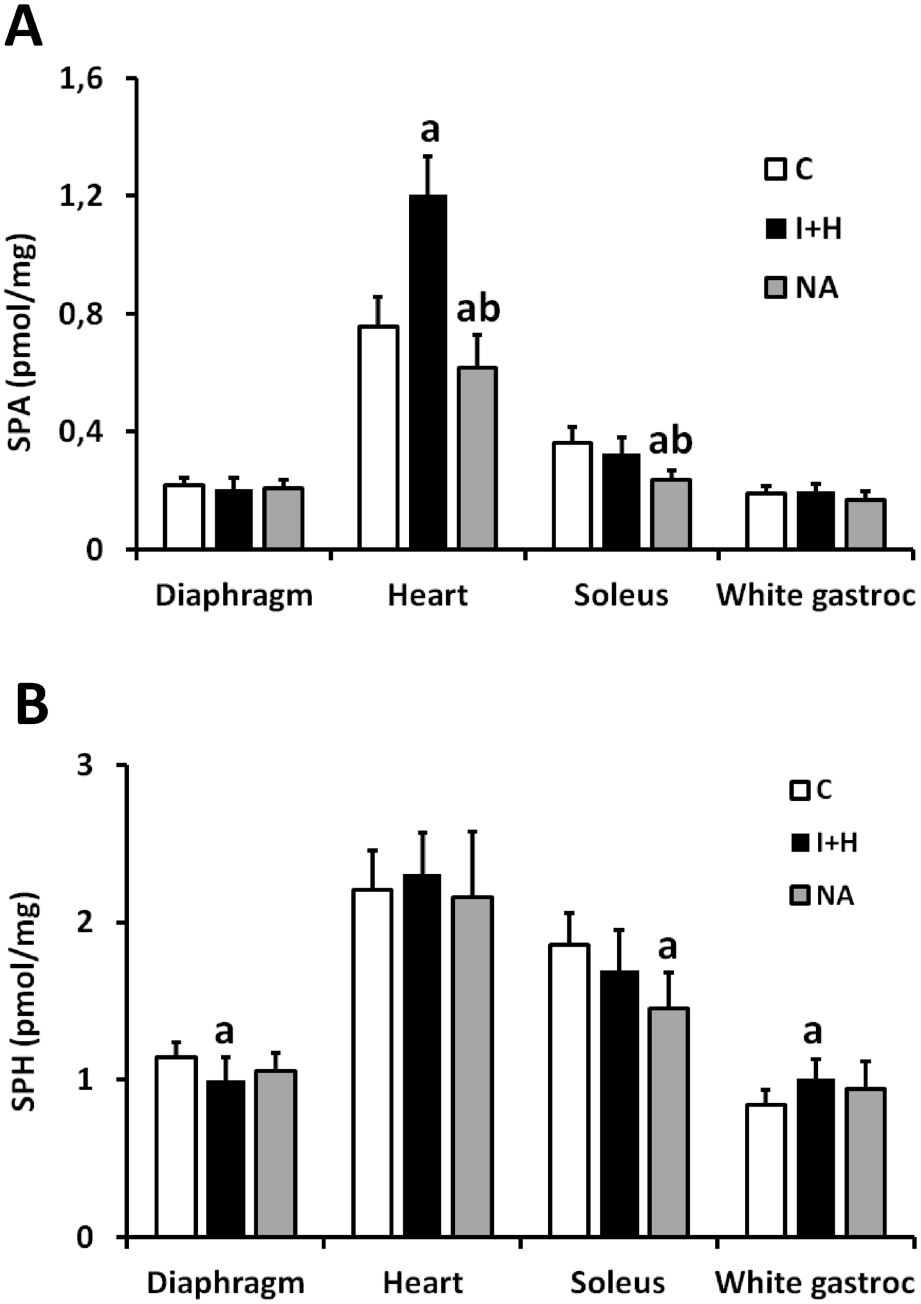
The impact of intralipid and heparin infusion or nicotinic acid infusion on the content of sphingoid bases in different rat muscles. Panel A—the content of sphinganine (SPA, n = 10); Panel B—the content of sphingosine (SPH, n = 10); C—control group; I+H—intralipid + heparin group; NA—nicotinic acid group. Values represent mean +/- SD; a—p<0.05 vs C, b—p<0.05 vs I+H.

The control level of sphinganine in the heart was more than two times higher than in any other muscle studied (Fig 5A). Regarding other muscle types, control level of sphinganine in soleus was significantly higher than in the diaphragm or white gastrocnemius (p<0.05). Heparin and intralipid treatment increased the sphinganine content only in the heart, whereas had no effect on its content in other muscle types. Nicotinic acid treatment had no impact on the content of sphinganine in diaphragm and white gastrocnemius, and lowered the content of the compound below control values in the heart and soleus muscle.

The control levels of sphingosine in the heart and in the soleus were higher than in other two muscles (Fig 5B). The reciprocal relations in the content of sphingosine were not affected by different treatments. Treatment with I+H or NA had only modest effect on the level of sphingosine. I+H reduced the level of the compound in the diaphragm and elevated it in the white gastrocnemius. Nicotinic acid reduced the level of sphingosine in the soleus.

### Correlation between ceramide FSR and the content of sphingolipids in different muscle types

In the diaphragm muscle Cer FSR displayed significant positive correlation only with C24:0-Cer (r = 0.57, p<0.0042, S1 Table). In the heart Cer FSR displayed strong positive correlation with sphinganine (r = 0.85, p<0.001), long-chain ceramides (C22:0-Cer, C24:0-Cer, C24:1-Cer, r>0.64, p<0.001) and the total content of ceramide (r = 0.77, p<0.001, S2 Table). In the soleus muscle Cer FSR displayed positive correlation with C18:0-Cer, C22:0-Cer, C24:1-Cer (r>0.51, p<0.004) and the total content of ceramide (r = 0.62, p<0.001, S3 Table), whereas in the white gastrocnemius with C16:0-Cer, C18:0-Cer, C20:0-Cer, C22:0-Cer (r>0.54, p<0.002) and total content of ceramide (r = 0.65, p<0.001, S4 Table).

### Correlation between plasma fatty acids and ceramides in different muscle types

In the diaphragm muscle total ceramide content and ceramide FSR was positively correlated with total plasma FFA and plasma FFA turnover (r>0.62, p<0.00025, S5 Table). At individual plasma fatty acids level, total diaphragm ceramide and Cer FSR showed positive correlation with C16:0-, C18:0-, C18:1- and C18:2-FFA (r>0.62, p<0.00025). At individual tissue ceramide level, only C22:0-Cer displayed positive correlation with total plasma FFA, plasma FFA turnover and some of the individual fatty acids (r>0.63, p<0.00025).

In the heart all of the individual plasma fatty acids (except C24:1-FFA), total plasma FFA and plasma FFA turnover rate displayed strong positive correlation with the content of sphinganine, total content of ceramide, Cer FSR and the content of C22:0-, C24:0- and C24:1-Cer (r>0.64, p<0.00025, S6 Table).

In the soleus muscle the individual plasma fatty acids (also except C24:1-FFA), total plasma FFA and plasma FFA turnover rate displayed strong positive correlation only with total content of ceramide, Cer FSR and C18:0-Cer (r>0.64, p<0.00025, S7 Table). The same could be noted for white gastrocnemius (r>0.66, p<0.00025 for the above parameters), with the addition of C16:0-Cer which correlated positively with most of the individual plasma fatty acids, total plasma FFA and plasma FFA turnover rate (r>0.66, p<0.00025, S8 Table).

## Discussion

The results obtained on plasma FFA metabolism clearly show that the procedures of elevation and reduction in the plasma free fatty acid concentration worked as expected. The changes in plasma fatty acids concentration were accompanied by respective change in whole body insulin sensitivity, as estimated by HOMA-IR. Treatment with intralipid and heparin increased the plasma FFA concentration, plasma FFA turnover and significantly decreased whole body insulin sensitivity. Treatment with nicotinic acid reduced each of the above parameters, and significantly improved insulin sensitivity. Interdependence between fatty acids supply, ceramide synthesis rate and skeletal muscle insulin sensitivity was extensively described in our previous works [26–28]. Our current paper focuses on differences in ceramide synthesis rate under varied plasma fatty acids supply in muscles which significantly differ in their oxidative capacity and function. The heart relies only on oxidative production of ATP and free fatty acids cover around 70% of energy substrates used at the basal state [29]. The diaphragm is composed of slow-twitch oxidative fibers (type I, around 40%), the fast-twitch oxidative-glycolytic fibers (type IIa, around 30%) and the fast-twitch glycolytic fibers (type IIx, around 40%) [30–33]. The soleus muscle is composed mostly of high oxidative fibers whereas the white section of the gastrocnemius is composed of glycolytic fibers [34, 35]. The diaphragm oxidative potential is located between the soleus and the heart, in spite of the mixed fiber composition [36]. The examined leg muscles maintained only the basal tone through the experiment, whereas both respiration and heart rate was at its resting pace. In the rat, at rest, the heart contracts at 330–480 beats/min, whereas the diaphragm performs about 80 contractions/min [37]. Metabolism of sphingolipids was very often studied in vivo in the heart (e.g. [38–42]) and the hind leg muscles (e.g. [43–46]). On the contrary, there are only few data concerning metabolism of the compounds in the diaphragm [47, 48]. The level of ceramide in the heart and in the leg muscles are similar to the data previously reported in the rat [16, 38]. The content of ceramide in the diaphragm in the present study is lower than reported by Empinado et al. [47]. However, the rats in the latter study were much older than our rats (15 weeks vs. 26 weeks) and this could contribute to the difference. At the basal state, the Cer FSR in heart and the diaphragm is much higher than in the leg muscles. The basal SPT activity in the heart (no data on SPT activity in the diaphragm are available) is similar to the enzyme activity in the leg muscles [49, 50]. It is highly likely that higher Cer FSR observed in continuously contracting muscle arises from their higher fatty acids flux. Basal level of sphinganine in diaphragm is much lower than in the heart and in the soleus, yet Cer FSR is similar in those muscles. It might indicate onthe much faster entry of the newly synthesized compound in further steps of ceramide synthesis in the diaphragm than in the other muscles. However, the Cer FSR in particular muscles is also not reflected by the level of ceramide. The ratio: total level of Ceramide/Ceramide FSR is: the heart- 1.3, the diaphragm—3.3, the soleus—2.7 and the white gastrocnemius- 2.2. Above ratios decrease slightly with H+I treatment and are mainly unchanged with NA treatment. It would suggest that in the heart ceramide enters other metabolic routes quicker than in the three skeletal muscles and that the process is accelerated by increase in plasma FFA. The muscles were exposed to the same plasma concentration of FFA. So a reason of so much higher level of ceramide in the diaphragm comparing to other muscles studied would suggest reduction in its breakdown in this particular muscle. The level of sphingosine, the only product of ceramide breakdown, is lower in the diaphragm than in the heart and in the soleus which seems to support such reasoning.

It was previously shown that acute, over 100%, increase in the plasma FFA level was accompanied by increase in the ceramide content only in the soleus but neither in the red nor in white section of the gastrocnemius [12]. In the intralipid-heparin group, the plasma FFA concentration increased several fold, which was comparable to the level observed in other infusion-based studies [51, 52], and during physical activity in men [53, 54]. However, it resulted only in mild, though significant elevation in the content of ceramide in each muscle studied. One could assume that exposure of the muscles for elevated plasma FFA supply should last much longer than in the present study (two hours) to induce considerable accumulation of ceramides in the tissues. Elevation in the content of ceramide in the obese subjects with elevated plasma FFA concentration and in isolated myocytes [6, 55] would favor such an option. However, the measurement of the fractional synthesis rate of ceramide indicates that it might be true in the skeletal muscles but not in case of the heart. In the heart, the Cer FSR was higher than the control value over 100% whereas in skeletal muscles it was elevated by around 50%. Interestingly, the level of sphinganine increased only in the heart. It would suggest that, in the heart the amount of sphinganine in this conditions exceeded the potential of its acylation to dihydroceramide, and that de-novo pathway of Cer synthesis dominates over salvage pathway in heart tissue. In the skeletal muscles, the *de novo* synthesized sphinganine was certainly used on further synthesis of ceramide, so no bottleneck effect was observed. The level of sphingosine remained stable in the heart and in the soleus which suggests stable rate of deacylation of ceramide in the experimental conditions. In I+H treated animals the ratio of total ceramide content/ Cer FSR was 2.3 in the diaphragm, 0.75 in the heart, 2.0 in the soleus and 3.7 in the white gastrocnemius. It would suggest that in the two contracting muscles newly synthesized ceramide was directed on the pathways of complex sphingolipid synthesis, thus high Cer FSR was not accompanied by respective increase in Cer concentration.

Treatment with NA reduced the plasma FFA concentration by about 50%. In spite of it, the *de novo* ceramide synthesis rate was significantly reduced only in the white gastrocnemius (glycolytic muscle) and only insignificantly in other examined muscles. It indicates that ceramide synthesis pathway in muscles with high oxidative potential is able to incorporate enough palmitate to assure proper supply of substrates for ceramide synthesis in the presence of reduced plasma free fatty acid supply. The drop in the ceramide content in the white gastrocnemius under decreased plasma fatty acids could be explained by its glycolytic metabolism and poor vascularization [56]. Moreover, the content of the fatty acid transporting proteins and thus a potential of the glycolytic muscle to take up fatty acids is much lower than of the red muscle or the heart [57]. In consequence, the amount of free fatty acids entering the white gastrocnemius myocytes is obviously reduced in NA-treated group. As a result, less plasma FFA could reach the ceramide synthesis pathway in the muscle. The content of sphinganine remained stable in the muscles with the exception of the soleus. It would be in line with the data on the synthesis rate. The difference between the content of sphinganine and the synthesis rate in the leg muscles are difficult to explain at present. The stable level of sphingosine in the two contracting muscle would suggest unchanged deacylation rate of ceramide. Again the level of the compound decreased in the soleus which would mirror the reduction in the content of ceramide in the muscle.

Finally, it has to be noted that the content of individual ceramide molecular species was affected differently under the varied supply of plasma FFA. In the diaphragm, only the very-long acyl-chain ceramides (C22:0- and C24:0) increased under the I+H treatment and only C24:0-Cer displayed positive correlation with Cer FSR. In the heart the same could be noted for most of the very-long chain ceramides such as C22:0-, C24:0- and C24:1-Cer. This indicate similar pathways of ceramide synthesis in continuously-contracting muscles, which prefers very long-chain fatty acids for sphinganine acylation. In soleus and white gastrocnemius increased supply in FFA affected C18:0-Cer and C16:0-Cer (in white gastrocnemius only). Both ceramides displayed also positive correlation with Cer FSR, which suggests preference for medium-chain fatty acids (especially stearate, C18:0-FFA) for sphingosine acylation. Above differences could be explained by the expression of different ceramide synthase isoforms in each of the studied muscles. In the heart CerS2 and CerS4 are the major ceramide synthase isoforms, whereas in skeletal muscle predominantly CerS1 and CerS5 isoforms are expressed [58, 59]. CerS2 and CerS4 displays substrate preference towards very long chain FFA (C22:0-FA and longer for CerS2 and C18:0-FA and longer for CerS4), whereas CerS1 and CerS5 show specificity towards stearate (C18:0-FA) and palmitate (C16:0-FA), which explains preference for very long-chain ceramide synthesis in the heart and long-chain ceramide synthesis in the skeletal muscle. The composition of CerS isoforms in diaphragm muscle has not been reported, but our data suggest that CerS6 could be the major isoform. The muscle Cer composition reflects also individual plasma FFA dynamics under intralipid/heparin infusion. The increase in plasma abundance of C16:0, C18:0 and C20:0 FA was 3–8 fold as compared with 20 fold increase in the case of 22:0 FA. Specific 22:0-Cer (and longer Cer’s) increased significantly in muscles which express appropriate CerS, but remained unaffected in soleus and white gastrocnemius which does not express CerS specific towards very-long chain fatty acids.

Taken together, we have shown that, at basal conditions, the ceramide fractional synthesis rate depends on the muscle type-it is higher in the heart and diaphragm than in the leg muscles. The fractional synthesis rate does not correspond with the level of ceramide in particular muscles. The differences in the content of ceramide between particular muscles cannot be solely explained by ceramide fractional synthesis rate, and are possibly affected by ceramide degradation and complex sphingolipid synthesis. In our opinion full depiction of fatty acid flow through sphingolipid molecules in such multi-variable environment requires development of novel methodologies to track FSR and concentration of not only ceramide but also complex sphingolipids.

## Supporting information

S1 Table Correlation between ceramide synthesis rate and the content of individual sphingolipid molecular species in rat diaphragm. (XLSX)

S2 Table Correlation between ceramide synthesis rate and the content of individual sphingolipid molecular species in rat heart. (XLSX)

S3 Table Correlation between ceramide synthesis rate and the content of individual sphingolipid molecular species in rat soleus muscle. (XLSX)

S4 Table Correlation between ceramide synthesis rate and the content of individual sphingolipid molecular species in rat white gastrocnemius muscle. (XLSX)

S5 Table Correlation between rat diaphragm sphingolipid composition and synthesis rate, plasma free fatty acids composition and appearance rate and whole-body insulin resistance (HOMA-IR). (XLSX)

S6 Table Correlation between rat heart sphingolipid composition and synthesis rate, plasma free fatty acids composition and appearance rate and whole-body insulin resistance (HOMA-IR). (XLSX)

S7 Table Correlation between rat soleus muscle sphingolipid composition and synthesis rate, plasma free fatty acids composition and appearance rate and whole-body insulin resistance (HOMA-IR). (XLSX)

S8 Table Correlation between rat white gastrocnemius muscle sphingolipid composition and synthesis rate, plasma free fatty acids composition and appearance rate and whole-body insulin resistance (HOMA-IR). (XLSX)
